# The value of C-reactive protein, leucocytes and vital signs in detecting major complications after oncological colorectal surgery

**DOI:** 10.1007/s00423-024-03266-3

**Published:** 2024-02-27

**Authors:** Anke H. C. Gielen, Maud Schoenmakers, Stephanie O. Breukink, Bjorn Winkens, Jischmaël van der Horst, Kevin P. Wevers, Jarno Melenhorst

**Affiliations:** 1 Department of Surgery, Maastricht University Medical Centre, P.O. Box 5800, 6202 AZ Maastricht, The Netherlands; 2 School of Nutrition and Translational Research in Metabolism (NUTRIM), Maastricht University, Maastricht, The Netherlands; 3 Faculty of Health, Medicine and Life Sciences, Maastricht University, Maastricht, The Netherlands; 4 GROW School for Oncology and Developmental Biology, Maastricht, The Netherlands; 5 Department of Methodology and Statistics, Care and Public Health Research Institute (CAPHRI), Maastricht University, Maastricht, The Netherlands

**Keywords:** Colorectal cancer, Postoperative monitoring, Complications, CRP, Leucocytes, Vital signs

## Abstract

**Purpose:**

To assess the association of postoperative C-reactive protein (CRP), leucocytes and vital signs in the first three postoperative days (PODs) with major complications after oncological colorectal resections in a tertiary referral centre for colorectal cancer in The Netherlands.

**Methods:**

A retrospective cohort study, including 594 consecutive patients who underwent an oncological colorectal resection at Maastricht University Medical Centre between January 2016 and December 2020. Descriptive analyses of patient characteristics were performed. Logistic regression models were used to assess associations of leucocytes, CRP and Modified Early Warning Score (MEWS) at PODs 1–3 with major complications. Receiver operating characteristic curve analyses were used to establish cut-off values for CRP.

**Results:**

A total of 364 (61.3%) patients have recovered without any postoperative complications, 134 (22.6%) patients have encountered minor complications and 96 (16.2%) developed major complications. CRP levels reached their peak on POD 2, with a mean value of 155 mg/L. This peak was significantly higher in patients with more advanced stages of disease and patients undergoing open procedures, regardless of complications*.* A cut-off value of 170 mg/L was established for CRP on POD 2 and 152 mg/L on POD 3. Leucocytes and MEWS also demonstrated a peak on POD 2 for patients with major complications.

**Conclusions:**

Statistically significant associations were found for CRP, Δ CRP, Δ leucocytes and MEWS with major complications on POD 2. Patients with CRP levels ≥ 170 mg/L on POD 2 should be carefully evaluated, as this may indicate an increased risk of developing major complications.

## Introduction

Colorectal cancer (CRC) is currently the third most frequent form of malignancy. The incidence is predicted to increase in the coming years with an estimation of over 2 million new cases diagnosed worldwide annually by 2030 [1, 2]. Despite the development of the ‘Wait and See’ protocol as one of the regular treatment options for rectal cancer, surgical intervention remains the cornerstone in primary CRC treatment [3].

Postoperative infectious complications, especially anastomotic leakage, are the most prevalent and concerning complications following colorectal resections with a primary anastomosis [4, 5]. It is well-established that anastomotic leakage and its sequelae not only lead to increased short-term morbidity and mortality, but also have a negative impact on long-term oncological outcomes such as disease-free and overall survival [6–9]. Thus, early detection and adequate management of these complications are crucial for improving patient outcomes.

Previous studies have identified numerous biomarkers assisting in early diagnosis of anastomotic leakage and other major complications [10, 11]. C-reactive protein (CRP) currently is the gold standard [12]. Several cut-off points on postoperative days 3, 4 and 5 have been suggested and have demonstrated good diagnostic accuracy [10, 13]. However, the rate of change or trajectory of CRP levels may be more significant than the cut-off point alone [12, 14]. As an acute phase protein, CRP is affected not only by potential complications but also by the biological stress of the surgical procedure itself and peaks on the second postoperative day in all patients, regardless of complications [15, 16], resulting in a significantly increased specificity reaching 91% for CRP on the third postoperative day, in contrast to a specificity of 57% on POD 2 [17]. There is no consensus on the added value of leucocytes in the detection of complications [12, 18, 19].

Entering a new era of minimally invasive colorectal surgery, even striving for ambulatory surgery or same day discharge, the way of adequately monitoring our postoperative patients will have to change accordingly [20, 21]. We are considering the feasibility of orchestrating outpatient laboratory tests for CRP and/or leucocytes, or providing patients with biosensor monitoring at home [22].

In view of these knowledge gaps, we aim to assess the association of postoperative C-reactive protein (CRP), leucocytes and vital signs in the first three postoperative days (PODs) with major complications after oncological colorectal resections in a tertiary referral centre for colorectal cancer in The Netherlands.

## Methods

### Study design

An observational cohort study was conducted of all patients undergoing oncological colorectal resections in the Maastricht University Medical Centre + , a tertiary referral centre for colorectal cancer in The Netherlands. Included patients were treated between 1 January 2016 and 31 December 2020. In this period, the standard protocol of postoperative care in our hospital included daily laboratory tests (including CRP and leucocytes) in this patient population.

All oncological colorectal resections were eligible for inclusion. Criteria for exclusion were an age under 18, a benign indication for the colorectal resection and surgical procedures without (segmental) bowel resection or essential missing data in the electronic patient record.

Data were collected retrospectively from the electronic patient record using CTcue software (v3.1.0, CTcue B.V., Amsterdam, The Netherlands). Recorded data regarding baseline characteristics included patient age, sex, body mass index (BMI), smoking status, comorbidities and American Society of Anaesthesiologists (ASA) classification [23]. Both open, laparoscopic and robot-assisted procedures were included. Recorded clinical parameters included indication for operation, acute or elective procedure, type of surgery, duration of surgery (in minutes), primary anastomosis (yes/no), CRP and leucocytes in the first five postoperative days, the Modified Early Warning Score (MEWS) [24] measured in the morning of the first five postoperative days, complications according to the Clavien-Dindo classification [25] and length of hospital stay. Minor complications were defined as Clavien-Dindo grade I and II, major complications as Clavien-Dindo ≥ grade III. Complications were promptly registered upon their occurrence and diagnosis. A systematic evaluation of all complications took place on the day following the patients’ discharge and thoroughly discussed by the entire colorectal surgical staff in a daily meeting. The study protocol was assessed and approved by the medical ethics committee of the Maastricht University Medical Centre + (protocol number 2022–3598).

### Statistical analysis

All statistical analyses were conducted in IBM SPSS Statistics (version 28: IBM Corporation, Armonk, NY, USA) after consulting a dedicated statistician. Clinical characteristics of the patients were described using mean and standard deviation (SD) or median and range (minimum–maximum) for numerical variables and number of patients with percentage for categorical ones. Cut-off points for the potential variables (CRP, leucocytes and MEWS) predictive for major complications were determined by receiver operating characteristic (ROC) curve. Optimal cut-off points were based on Youden’s J index, i.e. maximizing sum of sensitivity and specificity. Logistic regression analyses were used to assess associations of CRP, leucocytes and MEWS with the occurrence of major postoperative complications, with correction for potential confounders. This was done using the data of the first three PODs, since most patients without complications were already discharged on POD 3, or discharged without new laboratory testing on POD 4 or 5. The confounders were defined prior to the analyses and included age (years), sex (male or female), stage of disease (I, II, III or IV), BMI, diabetes mellitus and active smoking. Two-sided *p*-values ≤ 0.05 were considered statistically significant.

## Results

In total, 594 patients were included. The population consisted of 340 (57.2%) male and 254 (42.8%) female patients with a mean age of 72.6 (SD 10.9) years (Table 1). Most patients underwent an oncological colorectal resection for CRC (*N* = 579, 97.5%), while four patients underwent the procedure for ovarian cancer, three for anal cancer, two for a lymphoma, two for a sarcoma, two for an appendiceal carcinoma, one for an intestinal melanoma and one for an endometrial carcinoma. Laparoscopic resections were performed in 353 (59.4%) cases, while robot-assisted procedures were performed in 22 (3.7%) cases (Table 2). In 64 (10.8%) cases, a primary laparoscopic procedure had to be converted to an open procedure. Primary open procedures were performed in 155 (26.1%) patients, predominantly in acute settings and in patients with locally advanced tumours. The majority of patients, 548 (92.3%), underwent an elective procedure, while 46 patients (7.7%) were operated on in an acute setting. The mean operating time was 210 (SD 92) minutes.

**Table 1:** Patient characteristics (*N* = 594)

Age in years, mean (SD^1^)	72.6 (10.9)
Sex, *N* (%)	
Male	340 (57.2%)
Female	254 (42.8%)
BMI^2^ (kg/m^2^), mean (SD)	26.6 (5.3)
Active smoking, *N* (%)	
Yes	91 (15.3%)
No	503 (84.7%)
Diabetes mellitus, *N* (%)	
Type I	3 (0.5%)
Type II	100 (16.8%)
No	491 (82.7%)
Cardiac history, *N* (%)	
Yes	61 (10.3%)
No	533 (89.7%)
Pulmonary history, *N* (%)	
Yes	67 (11.3%)
No	527 (88.7%)
ASA^3^ classification, *N* (%)	
I	48 (8.1%)
II	345 (58.1%)
III	193 (32.5%)
IV	8 (1.3%)
Operation indication, *N* (%)	
Colorectal cancer	579 (97.5%)
Local recurrence	5 (0.8%)
Anal cancer	3 (0.5%)
Ovarian cancer	4 (0.7%)
Other^4^	8 (1.3%)
Stage of disease^5^, *N* (%)	
I	178 (30.0%)
II	181 (30.5%)
III	180 (30.3%)
IV	35 (5.9%)

^1^*SD* standard deviation

^2^*BMI* body mass index

^3^*ASA* American Society of Anesthesiologists

^4^Indications classified as ‘other’: lymphoma (*n* = 2), sarcoma (*n* = 2), melanoma (*n* = 1), appendiceal carcinoma (*n* = 2), endometrial carcinoma (*n* = 1)

^5^Only applicable for primary tumours, colorectal cancer

**Table 2:** Surgical details (*N* = 5 94)

Surgical procedure, *N* (%)	
Ileocecal resection	2 (0.3%)
Right hemicolectomy	199 (33.5%)
Left hemicolectomy	46 (7.7%)
Sigmoid resection	117 (19.7%)
LAR^1^	99 (16.6%)
APR^2^	70 (11.8%)
TME^3^	25 (4.2%)
Exenteration	12 (2.0%)
Other^4^	24 (4.0%)
Priority of procedure, *N* (%)	
Acute	46 (7.7%)
Elective	548 (92.3%)
Type of procedure, *N* (%)	
Laparoscopic	353 (59.4%)
Robot (assisted)	22 (3.7%)
Conversion	64 (10.8%)
Open	155 (26.1%)
Operative time in minutes, mean (SD^5^)	210 (92)
Primary anastomosis, *N* (%)	419 (70.5%)
Stoma, *N* (%)	
Ileostomy	93 (15.7%)
Colostomy	154 (25.9%)
Stoma prior to resection	12 (2.0%)
Postoperative stay (*d*), (range)	7 (3–77)

^1^*LAR* low anterior resection

^2^*APR* abdominoperineal resection

^3^*TME* total mesorectal excision

^4^The following procedures were classified as ‘other’: transversectomy (*n* = 8), (sub)total colectomy (*n* = 9), right hemicolectomy combined with sigmoid resection (*n* = 5), right hemicolectomy combined with APR (*n* = 1), pelvic exenteration paired with right hemicolectomy (*n* = 1)

^5^*SD* standard deviation

### Postoperative complications

The occurrence of postoperative complications is presented in Table 3. Of the 594 patients, 364 (61.3%) had recovered without any postoperative complications, 134 (22.6%) encountered minor (wound) complications and 96 (16.2%) suffered from major complications. Three patients (0.5%) died within 30 days after the procedure due to postoperative complications (Table 4). Among the 443 patients with a primary anastomosis in situ, 27 (6.1%) developed an anastomotic leakage. Out of these patients, 23 required re-operation. The remaining four patients were successfully managed conservatively through drainage and/or antibiotic therapy. The most frequently reported surgical complications were ileus (*N* = 89, 15%), an intra-abdominal abscess (*N* = 34, 5.7%) and surgical site infection (*N* = 29, 4.9%). Thirty-five (5.9%) patients had to be readmitted to the hospital within 30 days after initial discharge. The median length of hospital stay was 7 days, ranging from 3 to 77 days. Fifteen patients (2.5%) were discharged to their homes on the third postoperative day (POD) without any complications, 83 patients (14%) were discharged on POD 4 and 101 patients (17%) on POD 5.

**Table 3:** Postoperative complications (*N* = 594)

No complications, *N* (%)	364 (61.3%)
*Surgical complications*, *N* (%)	
Anastomotic leakage^1^	27 (6.1%)
Re-operation	23 (3.9%)
Intra-abdominal abscess	34 (5.7%)
Ileus	89 (15.0%)
Surgical site infection	29 (4.9%)
Fascia dehiscence	11 (1.9%)
High output stoma	14 (2.4%)
Bleeding	14 (2.4%)
*General complications*, *N* (%)	
Urinary tract infection	20 (3.4%)
Bladder retention	19 (3.2%)
Central line infection	6 (1.0%)
Pneumonia	40 (6.7%)
Thrombo-embolic events^2^	9 (1.5%)
Delirium	10 (1.7%)
Readmissions to hospital	35 (5.9%)
Mortality	3 (0.5%)
Other^3^	18 (3.0%)

^1^Only patients who had an anastomosis were considered for the percentage of anastomotic leakages

^2^Complications classified as thrombo-embolic events were pulmonary embolism (*n* = 5), deep venous thrombosis (*n* = 2), arterial thrombosis (*n* = 1) and cerebrovascular accident (*n* = 1)

^3^Complications classified as ‘other’ were pancreatic leakage (*n* = 5), hypertension (*n* = 3), electrolyte disturbances (*n* = 2), perioperative injury of the urinary tract (*n* = 2), abdominal compartment syndrome (*n* = 1), intestinal ischemia (*n* = 1), burn injury (*n* = 1), hearing loss (*n* = 1), atelectasis (*n* = 1) and airway obstruction (*n* = 1)

**Table 4:** Complication classification (*N* = 594)

Clavien-Dindo classification, *N* (%)	
No complication	364 (61.3%)
Grade I	40 (6.7%)
Grade II	94 (15.8%)
Grade IIIa	33 (5.6%)
Grade IIIb	30 (5.1%)
Grade IV	26 (4.4%)
Grade IVa	4 (0.7%)
Grade IVb	0
Grade V	3 (0.5%)

### C-reactive protein (CRP)

**Fig. 1:**
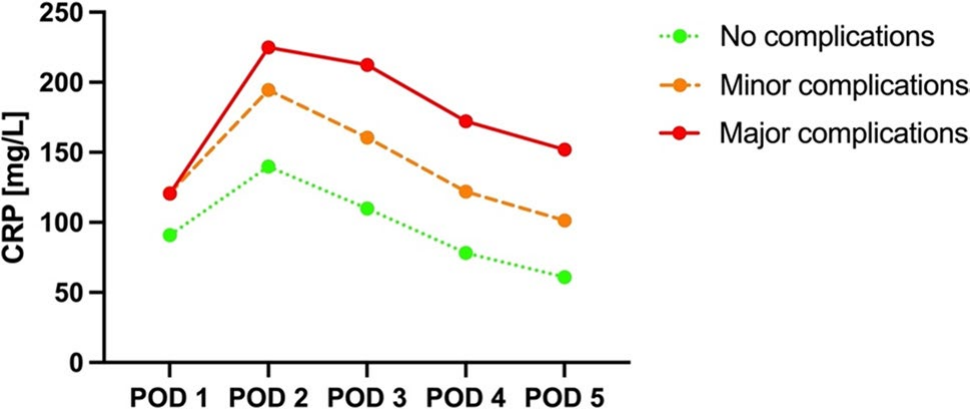
Mean CRP levels with regard to complications

CRP levels reach their peak on the POD 2, with a mean value of 166 mg/L (SD 88.6), gradually decreasing over the subsequent days. The data were stratified to analyse the CRP levels in patients with no, minor and major complications (Fig. 1). The peak CRP levels on POD 2 were 140 mg/L, 195 mg/L and 225 mg/L for patients without complications, with minor and with major complications, respectively.

**Fig. 2:**
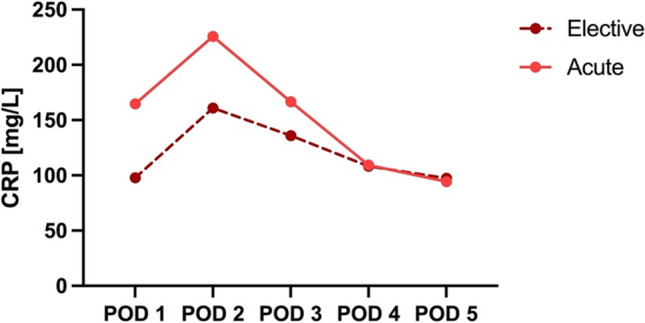
Mean CRP levels for acute and elective procedures per postoperative day

Figure 2 displays the mean CRP levels for acute and elective procedures on each postoperative day. Mean CRP peaked for both acute and elective procedures on POD 2 to 226 mg/L and 161 mg/L, respectively. Patients undergoing acute procedures had higher mean CRP levels on PODs 1, 2 and 3 compared to patients undergoing elective procedures; however, we must report that most acute cases were operated by laparotomy.

**Fig. 3:**
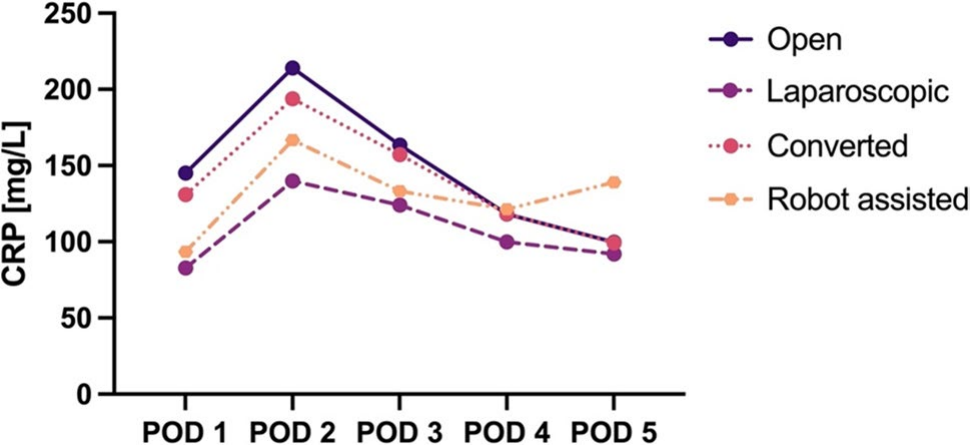
Mean CRP per type of surgical procedure

The mean CRP per surgical procedure type is presented in Fig. 3. All procedure types demonstrated a peak in mean CRP levels on POD 2, with primary open procedures showing the highest peak at 214 mg/L, followed by converted procedures at 194 mg/L, robot-assisted procedures at 167 mg/L and laparoscopic procedures with the lowest peak at 140 mg/L. It should be noted that all robot-assisted procedures were rectal resections.

**Fig. 4:**
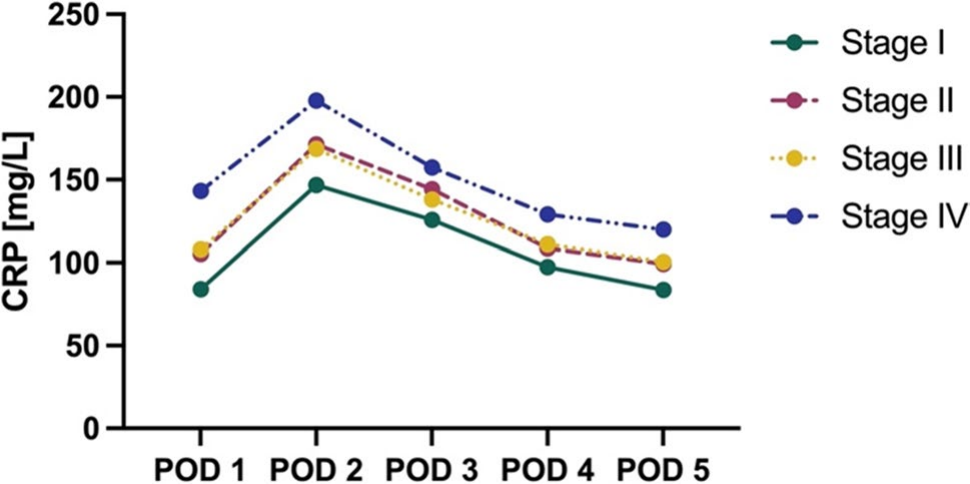
Mean CRP per clinical stage of disease

When stratified by clinical stage of disease, CRP levels displayed a significantly higher peak on POD 2 for patients with stage IV disease, with a mean CRP of 198 mg/L. Stages II and III exhibited similar peak values of 171 mg/L and 169 mg/L on POD 2, respectively. Stage I patients demonstrated the lowest peak at 147 mg/L (Fig. 4).

Receiver operating characteristic (ROC) curve analysis was performed for all patients on POD 2 and POD 3. The optimal cut-off was determined using Youden’s J index. On POD 2, the established cut-off was 170 mg/L with a sensitivity of 68% and specificity of 62%. Notably, the specificity increased on POD 3, with an established cut-off of 152 mg/L, sensitivity 69% and specificity 74% (Table 5). If T4 tumors, open and acute resections were excluded, the area under the curve (AUC) would be even higher.

**Table 5:** ROC curve analysis for POD 2 and POD 3 to assess cut-off values for CRP as a predictor for major complications

					95% CI^1^	
	CRP cut-off (mg/L)	Sensitivity	Specificity	AUC^2^	Lower bound	Upper bound
POD 2	170	0.68	0.62	0.71	0.65	0.77
POD 3	152	0.69	0.74	0.74	0.69	0.81

*CRP* C-reactive protein, *POD* postoperative day

^1^*CI* confidence interval

^2^*AUC* area under the curve

### Leucocytes

**Fig. 5:**
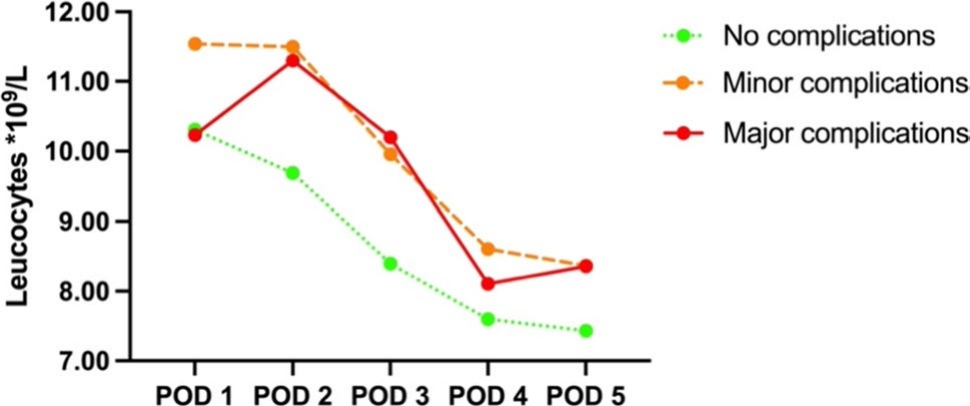
Mean leucocytes with regard to complications

The leucocyte levels also demonstrate a peak on POD 2 for patients with minor or major complications (Fig. 5). For these patient groups, leucocyte levels reached a peak of 11.5 10^9/L and 11.3 10^9/L*,* respectively. Leucocyte levels gradually decreased over the subsequent days in a pattern comparable to the CRP levels. In patients without complications, the level of leucocytes gradually decreased from POD 1 onwards.

### Modified Early Warning Score (MEWS)

The MEWS values for each postoperative day (POD) ranged from 0 to 10, with a mean MEWS variation of 0.5–1.5 during the initial five postoperative days. We acknowledge that these minor differences may not have significant clinical relevance.

### Logistic regression analyses

Confounder-adjusted logistic regression analyses were performed on the first three postoperative days, since most patients without complications were discharged on POD 3. The results of these analyses are depicted in Table 6. A statistically significant association was observed between CRP levels and major complications on PODs 1, 2 and 3 (*p* = 0.03, *p* = 0.03 and *p* < 0.001, respectively). The same association was demonstrated for the change in CRP levels compared to the preceding day (Δ CRP) on POD 2 and 3 (*p* < 0.001). Leucocyte levels on POD 3 showed a statistically significant association with major complications (*p* = 0.003), while the Δ leucocytes was only significant on POD 2 (*p* < 0.001). The association between MEWS and major complications was only statistically significant on POD 2 (*p* = 0.02).

**Table 6:** Multivariable logistic regression analyses assessing the associations of (delta) CRP, (delta) leucocytes and MEWS, measured on POD 1, 2 or 3, with major postoperative complications

	POD 1			POD 2			POD 3		
	OR	95% CI	*p*	OR	95% CI	*p*	OR	95% CI	*p*
CRP	1.01	1–1.01	**0.03**	1.01	1–1.01	**0.03**	1.01	1.01–1.012	** < 0.001**
Δ CRP^1^	N/A	N/A	N/A	1.01	1.01–1.02	** < 0.001**	1.01	1.01–1.02	** < 0.001**
Leucocytes	0.96	0.89–1.03	0.26	1.00	0.92–1.09	0.98	1.11	1.04–1.19	**0.003**
Δ Leucocytes^2^	N/A	N/A	N/A	1.27	1.13–1.43	** < 0.001**	1.03	0.92–1.15	0.62
MEWS	1.10	0.78–1.54	0.59	1.21	1.03–1.42	**0.02**	1.20	0.99–1.44	0.06

^1^Delta CRP on POD 2 equals CRP on POD 2 minus CRP on POD 1

Delta CRP on POD 3 equals CRP on POD 3 minus CRP on POD 2

^2^Delta leucocytes on POD 2 equals leucocytes on POD 2 minus leucocytes on POD 1

Delta leucocytes on POD 3 equals leucocytes on POD 3 minus leucocytes on POD 2

*CI* confidence interval, *CRP* C-reactive protein, *POD* postoperative day, *N/A* not applicable

Depicting the primary model, with correction for sex, age, stage of disease, BMI, diabetes mellitus and active smoking. Statistically significant results were defined as *p* ≤ 0.05 and are printed in bold

## Discussion

In this study, we analysed the levels of postoperative CRP, leucocytes and vital signs in 594 consecutive patients who underwent oncological colorectal resections. Our findings support that postoperative CRP levels can serve as a predictive factor for major complications. Specifically, we identified cut-off values of 170 mg/L on POD 2 and 152 mg/L on POD 3. Furthermore, we found a statistically significant association between changes in both CRP and leucocyte levels and MEWS on POD 2 with the occurrence of major complications.

While previous research has primarily focused on establishing cut-off values for POD 3, only a limited number of studies have proposed cut-off values for POD 2 [11, 13, 19]. However, no consensus has been reached on the optimal cut-off point for any postoperative day. The suggested CRP values in available literature range from 140 to 220 mg/L [10, 11, 16, 26]. Our identified cut-off value of 152 mg/L on POD 3 falls within this range. Moreover, other studies have demonstrated that an increase of ≥ 50 mg/L between any two postoperative days, irrespective of the absolute CRP value, predicts the occurrence of complications [12, 17].

CRP levels are higher in patients who undergo acute resections. Open resections are more frequently performed in an acute setting, which contributes to the observed elevation in CRP levels. This is partly due to the inflammatory response in the indication for surgery (i.e. near blowout, perforation or peritonitis), and partly contributed to the fact that open surgery generates higher CRP levels than minimally invasive procedures [27, 28]. In our study population, patients who underwent robotic resections exhibited slightly higher CRP levels than those who underwent laparoscopic procedures. Additionally, CRP levels further increased on POD 5 in the robotic group. This finding can be partially attributed to the longer operating time in the robotic group, as well as the fact that only rectal resections (no colon resections) were performed [29]. The second peak in CRP levels on POD 5 may be attributed to the fact that only patients who experienced complications remained hospitalized on day 5 after a robotic resection.

A more advanced clinical stage of disease is correlated with higher levels of postoperative serum CRP. This association has been demonstrated to be significant in relation to larger tumour diameter, therefore more open resections (including extra-anatomical resections due to T4 stage), longer operation time and more blood loss [30]. Several studies have also indicated that elevated postoperative CRP levels are significantly associated with long-term outcomes such as disease-free and overall survival, independent of complications [31–33].

Limited research has been conducted on the added value of the white blood cell count (WBC) or vital signs in predicting complications following colorectal surgery. Some studies suggest that WBC is not an independent risk for factor major complications, but becomes relevant when combined with elevated CRP levels [18]. Others argue that the addition of WBC to CRP does not improve the negative predictive value for anastomotic leakage, or that the trajectory of WBC over a 5-day period should be given more importance [12, 34]. Our findings suggest that the absolute WBC count on POD 2 may not have a significant association with major complications, but the change in WBC compared to POD 1 does exhibit a statistically significant association.

Reports on the value of vital signs in detecting complications vary considerably. Some studies report that vital sign abnormalities are very common after major abdominal surgery and therefore poor predictors of complications such as anastomotic leakage [35, 36]. Others argue that vital signs, particularly heart rate, respiratory rate and body temperature are associated with early detection of complications [37, 38]. Our study found that the MEWS, which incorporates heart rate, respiratory rate, temperature and blood pressure, exhibits a significant association with major complications when measured on POD 2 [24].

Certain limitations of the present study have to be acknowledged, including its retrospective design. Furthermore, no external validation has been conducted. The observed biases in the composition of study groups, where all robot-assisted procedures were rectal resections—typically elective and performed by experienced surgeons—while primary open procedures were predominantly performed in acute settings and on patients with locally advanced tumours, represent inherent limitations that may influence the interpretation of the results. These disparities introduce potential confounding factors, impacting postoperative CRP and leucocyte levels irrespective of postoperative complications. Nevertheless, the study’s substantial population size and adequate correction for confounding factors enhance its power and contribute valuable insights into the relationships examined.

Future prospective research is warranted to not only identify the optimal toolkit for early detection of postoperative complications but also ushering us into a new era of ambulatory colon resections and same day discharge, while ensuring adequate patient monitoring and treatment.

## Conclusion

CRP on POD 3, as previously established, serves as a predictive factor for major complications following oncological colorectal resections. However, the value on POD 2 is also highly indicative, which is of significant importance in an era where there is a trend towards 48- or even 24-h admissions for colorectal surgery. Particularly in uncomplicated, laparoscopic colorectal resections that have followed standard resection planes, a CRP above the cut-off value of 170 mg/L for CRP on POD 2, or ≥ 152 mg/L on POD 3 warrants vigilant patient monitoring.

## Data Availability

Data available on request from the authors.

## References

[1] Ferlay J, Soerjomataram I, Ervik M, Dikshit R, Eser S, Mathers C, Rebelo M, Parkin DM, Forman D, Bray F (2013) GLOBOCAN 2012 v1.0. Cancer incidence and mortality worldwide: IARC CancerBase 11 10.1002/ijc.29210 25220842

[2] Arnold M, Sierra MS, Laversanne M, Soerjomataram I, Jemal A, Bray F (2017). Global patterns and trends in colorectal cancer incidence and mortality. Gut.

[3] Beets-Tan R, Leijtens J, Beets GL (2011). Wait-and-see policy for clinical complete responders after chemoradiation for rectal cancer. Clin Oncol.

[4] Chiarello MM, Fransvea P, Cariati M, Adams NJ, Bianchi V, Brisinda G (2022) Anastomotic leakage in colorectal cancer surgery. Surg Oncol 40:101708 10.1016/j.suronc.2022.101708 35092916

[5] Kirchhoff P, Clavien P-A, Hahnloser D (2010). Complications in colorectal surgery: risk factors and preventive strategies. Patient Saf Surg.

[6] Bertelsen CA, Andreasen A, Jørgensen T, Harling H, Group DCC (2010). Anastomotic leakage after curative anterior resection for rectal cancer: short and long-term outcome. Colorectal Dis..

[7] Foppa C, Ng SC, Montorsi M, Spinelli A (2020). Anastomotic leak in colorectal cancer patients: new insights and perspectives. Eur J Surg Oncol.

[8] Koedam TW, Bootsma BT, Deijen CL, van de Brug T, Kazemier G, Cuesta MA (2022). Oncological outcomes after anastomotic leakage after surgery for colon or rectal cancer: increased risk of local recurrence. Ann Surg.

[9] Mirnezami A, Mirnezami R, Chandrakumaran K, Sasapu K, Sagar P, Finan P (2011). Increased local recurrence and reduced survival from colorectal cancer following anastomotic leak: systematic review and meta-analysis. Ann Surg.

[10] Singh P, Zeng I, Srinivasa S, Lemanu D, Connolly A, Hill A (2014). Systematic review and meta-analysis of use of serum C-reactive protein levels to predict anastomotic leak after colorectal surgery. J Br Surg.

[11] Messias BA, Botelho RV, Saad SS, Mocchetti ER, Turke KC, Waisberg J (2020). Serum C-reactive protein is a useful marker to exclude anastomotic leakage after colorectal surgery. Sci Rep.

[12] Smith SR, Pockney P, Holmes R, Doig F, Attia J, Holliday E (2018). Biomarkers and anastomotic leakage in colorectal surgery: C-reactive protein trajectory is the gold standard. ANZ J Surg.

[13] Straatman J, Harmsen AM, Cuesta MA, Berkhof J, Jansma EP, Van der Peet DL (2015). Predictive value of C-reactive protein for major complications after major abdominal surgery: a systematic review and pooled-analysis. PLoS ONE.

[14] Gans SL, Atema JJ, Van Dieren S, Koerkamp BG, Boermeester MA (2015). Diagnostic value of C-reactive protein to rule out infectious complications after major abdominal surgery: a systematic review and meta-analysis. Int J Colorectal Dis.

[15] Cole DS, Watts A, Scott-Coombes D, Avades T (2008). Clinical utility of peri-operative C-reactive protein testing in general surgery. Ann R Coll Surg Engl.

[16] Straatman J, de Weerdesteijn EdW, Tuynman JB, Cuesta MA, van der Peet DL (2016). C-reactive protein as a marker for postoperative complications. Are there differences in emergency and elective colorectal surgery?. Dis Colon Rectum..

[17] Stephensen B, Reid F, Shaikh S, Carroll R, Smith S, Pockney P (2020). C-reactive protein trajectory to predict colorectal anastomotic leak: PREDICT Study. J Br Surg.

[18] Liesenfeld LF, Sauer P, Diener MK, Hinz U, Schmidt T, Müller-Stich BP (2020). Prognostic value of inflammatory markers for detecting anastomotic leakage after esophageal resection. BMC Surg.

[19] Warschkow R, Beutner U, Steffen T, Müller SA, Schmied BM, Güller U (2012). Safe and early discharge after colorectal surgery due to C-reactive protein: a diagnostic meta-analysis of 1832 patients. Ann Surg.

[20] Tweed TT, Sier MA, Daher I, Bakens MJ, Nel J, Bouvy ND (2022). Accelerated 23-h enhanced recovery protocol for colon surgery: the CHASE-study. Sci Rep.

[21] Gignoux B, Gosgnach M, Lanz T, Vulliez A, Blanchet M-C, Frering V (2019). Short-term outcomes of ambulatory colectomy for 157 consecutive patients. Ann Surg.

[22] Kant N, Peters GM, Voorthuis BJ, Groothuis-Oudshoorn CG, Koning MV, Witteman BP (2022). Continuous vital sign monitoring using a wearable patch sensor in obese patients: a validation study in a clinical setting. J Clin Monit Comput.

[23] Doyle DJ, Goyal A, Bansal P, Garmon EH (2021). American Society of Anesthesiologists classification.

[24] Subbe CP, Kruger M, Rutherford P, Gemmel L (2001). Validation of a modified Early Warning Score in medical admissions. QJM.

[25] Dindo D, Demartines N, Clavien P-A (2004). Classification of surgical complications: a new proposal with evaluation in a cohort of 6336 patients and results of a survey. Ann Surg.

[26] Plat VD, Voeten DM, Daams F, van der Peet DL, Straatman J (2021). C-reactive protein after major abdominal surgery in daily practice. Surgery.

[27] Shibata J, Ishihara S, Tada N, Kawai K, Tsuno N, Yamaguchi H (2015). Surgical stress response after colorectal resection: a comparison of robotic, laparoscopic, and open surgery. Tech Coloproctol.

[28] Pedrazzani C, Moro M, Mantovani G, Lazzarini E, Conci S, Ruzzenente A (2017). C-reactive protein as early predictor of complications after minimally invasive colorectal resection. J Surg Res.

[29] Corbellini C, Biffi R, Luca F, Chiappa A, Costa S, Bertani E (2016). Open, laparoscopic, and robotic surgery for rectal cancer: medium-term comparative outcomes from a multicenter study. Tumori J.

[30] Shibutani M, Okazaki Y, Maeda K, HirakawA K, Ohira M (2021). A high postoperative serum C-reactive protein level has a negative impact on long-term survival, regardless of postoperative infectious complications, in patients who undergo laparoscopic surgery for colorectal cancer. Anticancer Res.

[31] McSorley ST, Watt DG, Horgan PG, McMillan DC (2016). Postoperative systemic inflammatory response, complication severity, and survival following surgery for colorectal cancer. Ann Surg Oncol.

[32] Matsubara D, Arita T, Nakanishi M, Kuriu Y, Murayama Y, Kudou M (2020). The impact of postoperative inflammation on recurrence in patients with colorectal cancer. Int J Clin Oncol.

[33] Yasui K, Shida D, Nakamura Y, Ahiko Y, Tsukamoto S, Kanemitsu Y (2021). Postoperative, but not preoperative, inflammation-based prognostic markers are prognostic factors in stage III colorectal cancer patients. Br J Cancer.

[34] El Zaher HA, Ghareeb WM, Fouad AM, Madbouly K, Fathy H, Vedin T (2022). Role of the triad of procalcitonin, C-reactive protein, and white blood cell count in the prediction of anastomotic leak following colorectal resections. World J Surg Oncol.

[35] Twohig K, Ajith A, Mayampurath A, Hyman N, Shogan BD (2021). Abnormal vital signs after laparoscopic colorectal surgery: more common than you think. Am J Surg.

[36] Erb L, Hyman NH, Osler T (2014). Abnormal vital signs are common after bowel resection and do not predict anastomotic leak. J Am Coll Surg.

[37] Luo J, Wu H, Jiang Y, Yang Y, Yuan J, Tong Q (2021). The role of heart rate, body temperature, and respiratory rate in predicting anastomotic leakage following surgery for rectal cancer. Mediat Inflamm.

[38] Den Dulk M, Witvliet M, Kortram K, Neijenhuis P, De Hingh I, Engel A (2013). The DULK (D utch leakage) and modified DULK score compared: actively seek the leak. Colorectal Dis.

